# Genotoxicity of selected cannabinoids in human lymphoblastoid TK6 cells

**DOI:** 10.1007/s00204-024-03826-y

**Published:** 2024-08-22

**Authors:** Nicol Kolar, Ezgi Eyluel Bankoglu, Helga Stopper

**Affiliations:** Institute of Pharmacology and Toxicology, University of Wuerzburg, 97078 Würzburg, Germany

**Keywords:** Cannabigerol, Cannabidiol, Cannabichromene, Cannabidivarin, Cannabinol, Genotoxicity, Micronuclei

## Abstract

Natural non-psychoactive cannabinoids such as cannabigerol (CBG), cannabidiol (CBD), cannabichromene (CBC), cannabidivarin (CBDV), and cannabinol (CBN) are increasingly consumed as constituents of dietary products because of the health benefits claims. Cannabinoids may reduce certain types of pain, nausea, and anxiety. Anti-inflammatory and even anti-carcinogenic properties have been discussed. However, there are insufficient data available regarding their potential (geno-)toxic effects. Therefore, we tested CBG, CBD, CBC, CBDV, and CBN for their genotoxic potential and effects on mitosis and cell cycle in human lymphoblastoid TK6 cells. The selected cannabinoids (except CBDV) induced increased micronuclei formation, which was reduced with the addition of a metabolic activation system (S9 mix). CBDV induced micronuclei only after metabolic activation. Mitotic disturbances were observed with all tested cannabinoids, while G1 phase accumulation of cells was observed for CBG, CBD and CBDV. The genotoxic effects occurred at about 1000-fold higher concentrations than are reported as blood levels from human consumption. However, the results clearly indicate a need for further research into the genotoxic effects of cannabinoids. The mechanism of the mitotic disturbance, the shape of the dose–response curves and the possible effects of mixtures of cannabinoids are aspects which need clarification.

## Introduction

Cannabinoids naturally occur in *Cannabis sativa*, liverworts, and fungi. In *Cannabis sativa*, more than 113 different cannabinoids have been identified so far, with the most abundant being tetrahydrocannabinol (THC), cannabidiol (CBD), cannabichromene (CBC), and cannabigerol (CBG) (Hanuš et al. 2016; Gülck and Møller 2020; Arif et al. 2021). Non-psychoactive cannabinoids have gained popularity in recent years because of their potentially beneficial effects on human health. Recently, the European Union (EU) has labelled cannabinoids and cannabinoid-containing extracts from *Cannabis sativa* as novel food. This means that such products will require pre-marketing authorization under the Novel Food Regulation (EU) 2015/2283. Until today, due to data gaps and uncertainties concerning potential genotoxicity and effects on gastrointestinal, nervous, and reproductive systems, the European Food Safety Authority (EFSA) has not concluded whether cannabinoids, more specifically CBD, are safe as food constituents (EFSA 2022; EMCDDA 2023). However, in the stores, hemp dietary products, which may contain non-psychoactive cannabinoids in varying amounts, can be found in the form of oils, capsules, and tinctures. These products can contain single or multiple cannabinoids, with cannabigerol (CBG), cannabidiol (CBD), cannabichromene (CBC), cannabidivarin (CBDV), and cannabinol (CBN) being frequently represented (Pavlovic et al. 2018; Calvi et al. 2018; Citti et al. 2019).

**Fig. 1:**
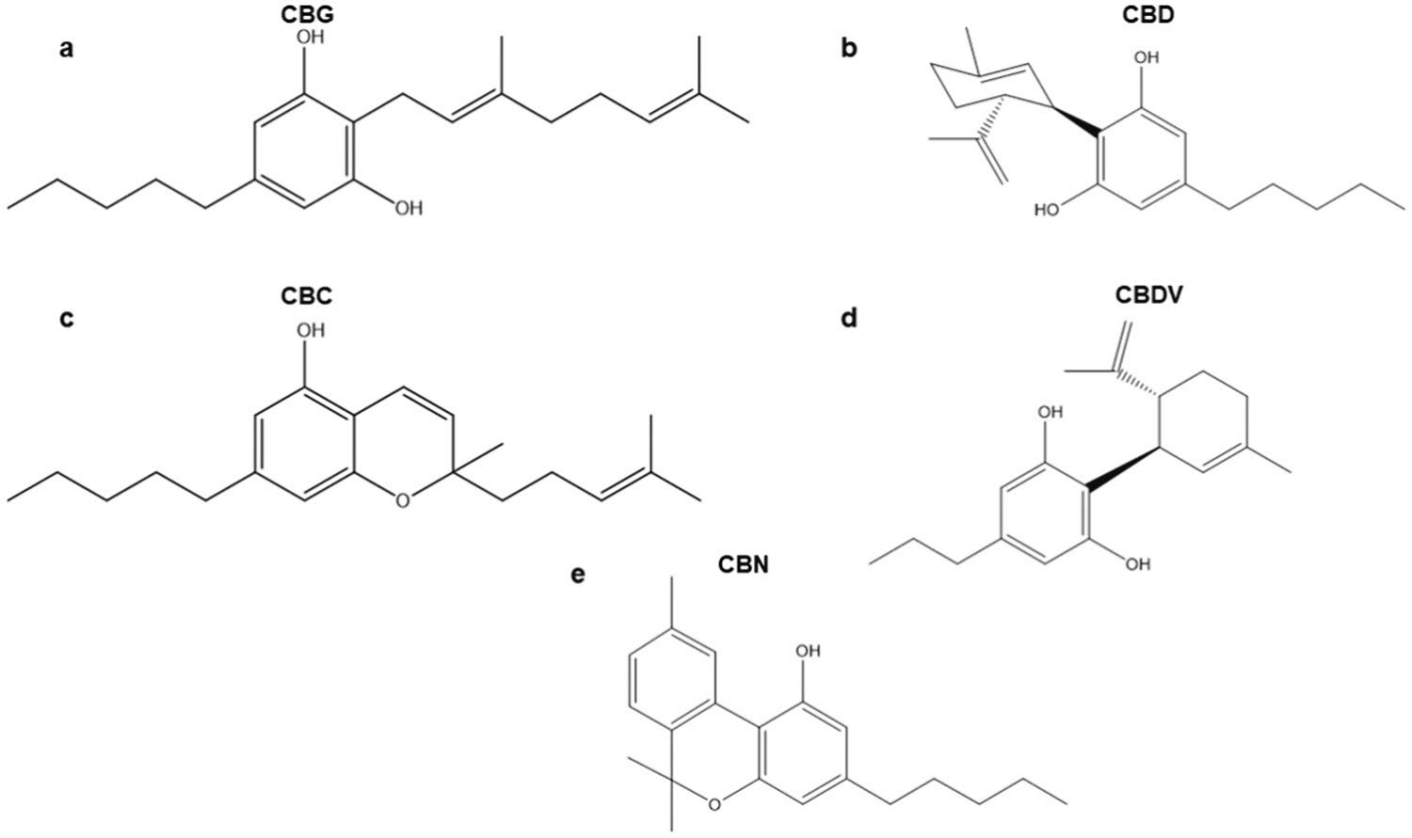
Chemical structures of cannabigerol (CBG) (**a**), cannabidiol (CBD) (**b**), cannabichromene (CBC) (**c**), cannabidivarin (CBDV) (**d**), and cannabinol (CBN) (**e**)

CBG, CBD, CBC, CBDV, and CBN are present in plant leaves, flowers, and seeds. They are structurally diverse: CBG is monocyclic, CBD, CBC, and CBDV are bicyclic, and CBN is a tricyclic cannabinoid (Fig. 1) (Chilakapati and Farris 2014; Tahir et al. 2021). Cannabinoids have been found to have certain antiepileptic, anticonvulsant, and antinociceptive properties (Anderson et al. 2021; Hurley et al. 2022; Kollipara et al. 2023; Arantes et al. 2024). Furthermore, it was suggested that they may also prevent inflammation and possibly be anti-carcinogenic (Abidi et al. 2022; Blevins et al. 2022; Gaweł-Bęben et al. 2023; Gojani et al. 2023). However, it was also reported that they have unclear genotoxic potential, cytotoxic effects, and reproductive toxicity (Russo et al. 2019; Cerretani et al. 2020; Gingrich et al. 2023; Li et al. 2023). The majority of the research findings are about CBD, while other cannabinoids (CBG, CBC, CBDV, and CBN) have been studied to a lesser extent.

The growing consumption of cannabinoids indicates the need to investigate them further. Therefore, in this study, we tested CBG, CBC, CBDV, and CBN, along with CBD, as single substances in vitro. We investigated the effects of these compounds in the micronucleus assay to obtain information about the genotoxic potential, monitored the presence of aberrant mitoses and performed kinetochore labelling, tubulin visualization and cell cycle analysis to gain some information about a possible mechanism. The compounds were tested in the human lymphoblastoid cell line TK6 with and without a metabolic activation system (S9 mix).

## Materials and methods

### Chemicals and reagents

Cannabigerol (CBG, CAS 25654-31-3, purity 99.6%), cannabidiol (CBD, CAS 13956-29-1, purity 98.7%), cannabichromene (CBC, CAS 20675–51-8, purity 99.6%), cannabidivarin (CBDV, CAS 24274-48-4, purity 98.3%), cannabinol (CBN, CAS 521-35-7, purity 99.0%) were obtained from LGC Standards (Augsburg, Germany). Dimethyl sulfoxide (DMSO, CAS 67–68-5) was from Carl Roth (Karlsruhe, Germany). RPMI 1640 medium (Cat. No. R5886), L-glutamine (CAS 56-85-9), sodium pyruvate (CAS 113-24-6), penicillin (100 µg/mL)/streptomycin (1 mM) (Cat. No. P0781), methyl methane sulfonate (MMS, CAS 66–27-3), methanol (CAS 67–56-1, ≥ 99.9%), ethanol (CAS 64-17-5, ≥ 99.9%), cytochalasin B (CAS 14930–96-2), 1,4-diazabicyclo[2.2.2]octane (DABCO, CAS 280-57-9), 4′,6-diamidino-2-phenylindole (DAPI, CAS 28718–90-3), bisbenzimide (Hoechst 33,258, CAS 23491–45-4), FITC-conjugated anti-human IgG secondary antibody (Cat. No. F0132), anti-α-tubulin-FITC monoclonal antibody (Cat. No. F2168), and Tween20 (CAS 9005-64-5) were from Sigma-Aldrich (Steinheim, Germany). Fetal calf serum (Cat. No. AC-SM-0190) was obtained from Anprotec (Bruckberg, Germany). Mutazyme™ S9 mix (Cat. No. 11-405L) was obtained from Trinova Biochem (Giessen, Germany). Vincristine sulphate (CAS 57-22-7) solution was from TEVA (Ulm, Germany). Primary anti-centromere antibody (Cat. No. 15-234) was procured from Antibodies Incorporated (Davis, USA).

Gel Green Nucleic Acid stain (Cat. No. 41003) was obtained from Biotium (Darmstadt, Germany).MACSQuant® reagents (Cat. No. 130-125-753) for flow cytometry were acquired from Miltenyi Biotec (Bergisch Gladbach, Germany).

### Cell line

Human lymphoblastoid cells (TK6) were obtained from Dr. W.J. Caspary, NIEHS, RTP, USA and cultured in RPMI 1640 medium supplemented with 10% (v/v) fetal calf serum, 1% (*w*/*v*) l-glutamine, 1% (*w*/*v*) sodium pyruvate and 0.4% (*w*/*v*) antibiotics (penicillin/streptomycin) in an incubator with 5% CO_2_ at 37 °C. Cells were sub-cultured three times per week. Cells were seeded in a 6-well plate with 3 mL of medium for each experiment.

### Cytokinesis‑block micronucleus (CBMN) assay

Cannabinoid treatment concentrations were chosen according to preliminary data, in the range from non-toxic to moderate toxicity, seen as a reduction of proliferation index. The cells were treated for 4 h with CBG: 5–35 µM, CBD: 5–25 µM, CBC: 5–15 µM, CBDV: 5–40 µM, and cannabinol (CBN): 5–20 µM. Methyl methane sulfonate (MMS) was used as a positive control in a final concentration of 40 µM. The solvent control was DMSO. After 4 h of treatment (following recommendations in the OECD test guideline No. 487) (OECD 2023), the culture medium was renewed, and cells were exposed for 24 h to the cytokinesis inhibitor cytochalasin B (3 μg/mL). Then, cells were harvested, and slides were prepared with cytospin centrifugation. The cells were fixed in ice-cold methanol for at least 2 h. The slides were dried, and Gel Green staining (1:100 dilution in bi-distilled water) was applied for 7 min in the dark. Slides were mounted using 1,4-diazabicyclo[2.2.2]octane (DABCO) and coverslips. The TK6 cells were also treated with CBG 30 µM, CBD 15 µM, CBC 15 µM, CBDV 40 µM, and CBN 15 µM for 4 h with or without the addition of Mutazyme™ 5% S9 mix. Mutazyme™ consists of PB/BNF-induced male Sprague Dawley rat liver S9, which was lyophilized with NADP, D-glucose-6-phosphate, MgCl_2_/KCl in pH 7.4 sodium phosphate buffer. The S9 mix was dissolved directly in the RPMI-1640 medium and applied at a final concentration of 0.25%. An additional positive control for S9 mix experiments was aflatoxin B1 (5 µM). After the treatment, cells were processed as described above. Each independent experiment was repeated three times. Slides were coded before evaluation. The evaluation was performed with a Nikon Eclipse TE2000 fluorescence microscope. Mononucleated, binucleated, multinucleated, mitotic, and apoptotic cells were evaluated in 1000 cells, micronuclei were scored in 1000 binucleated cells on two slides each, and the mean was calculated. The cytokinesis-block proliferation index (CBPI) was calculated with the following formula:

$$\frac{1\times \text{No}.\text{ of mononuclear cells}+2\times \text{No}.\text{ of binuclear cells}+3\times \text{No}.\text{ of multinuclear cells}}{\text{No}.\text{ of mononuclear cells }+\text{No}.\text{ of binuclear cells}+\text{No}.\text{ of multinuclear cells}}.$$

### Mitotic disturbance assay

The cells were treated for 6 h with CBG 30 µM, CBD 15 µM, CBC 15 µM, CBDV 40 µM, and CBN 15 µM. The positive control was vincristine sulphate 0.0121 µM, and the solvent control was DMSO. For the experiments with or without the S9 mix (final concentration 0.25%), TK6 cells were treated with either CBDV 40 µM or vincristine sulphate 10 ng/mL for 6 h. DMSO with or without S9 mix was used as control. Further procedure was as described for micronucleus experiments, except with direct harvest after cannabinoid treatment without the incubation period with cytochalasin B. Two slides were evaluated with a Nikon Eclipse 55i fluorescent microscope for each experimental condition. The independent experiments were conducted three times.

**Fig. 2:**
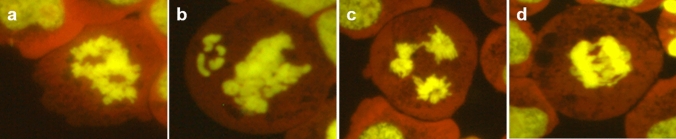
Representative images of disturbed metaphase (**a**, **b**) and anaphase-telophase (**c**, **d**) in TK6 cells. The cells were stained with Gel Green

The cells were counted in categories apoptosis, interphase, prophase, metaphase, anaphase-telophase, disturbed metaphase, and disturbed anaphase-telophase in 1000 cells. The mitotic index was calculated as a sum of the number of cells in prophase, metaphase, anaphase-telophase, disturbed metaphase, and disturbed anaphase-telophase in 1000 cells. Disturbed metaphase and disturbed anaphase-telophase were defined as deviating from the usual appearance of mitoses. Disturbed mitoses were assessed in 100 mitotic cells per slide. Representative sample images of mitotic disturbances are shown in Fig. 2.

### Kinetochore analysis of micronuclei

**Fig. 3:**
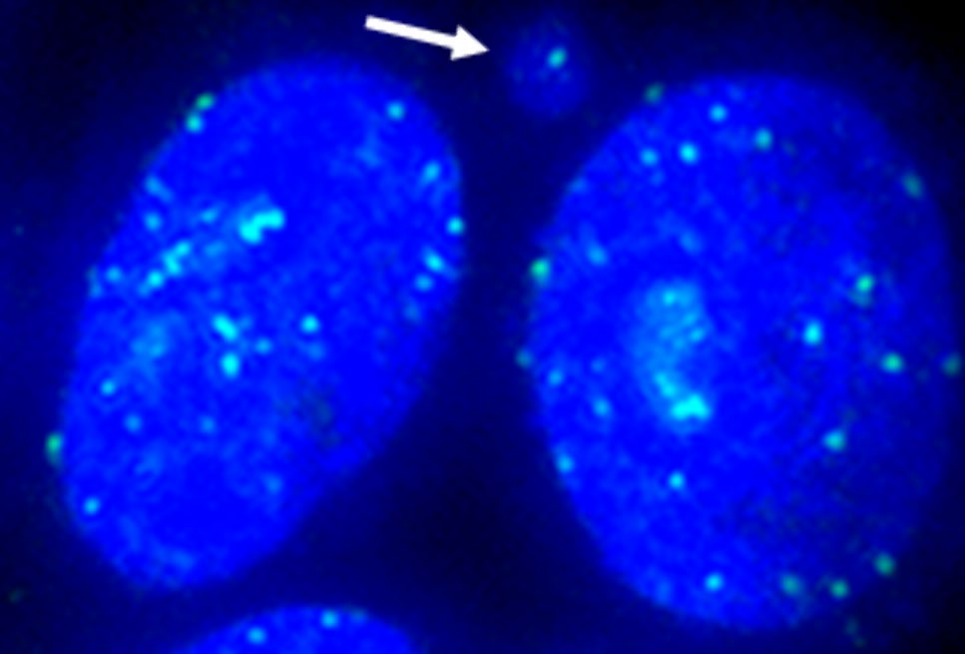
Representative image of kinetochore analysis. Nuclei (blue) were stained with Hoechst 33,258, and kinetochores (green) with primary anti-centromere and FITC-conjugated secondary antibodies. The arrow indicates the micronucleus with the kinetochore signal

For kinetochore analysis, cells were treated for 4 h with CBG 30 µM, CBD 15 µM, CBC 15 µM, CBDV 40 µM, and CBN 15 µM. The positive controls were MMS 40 µM, and vincristine sulphate 0.0121 µM; the solvent control was DMSO. After 4 h of treatment, the culture medium was renewed, and cells were exposed for 24 h to the cytokinesis inhibitor cytochalasin B (3 μg/mL). Then, cells were harvested, and slides were prepared with cytospin centrifugation. The cells were fixed in ice-cold methanol for 2 h. Kinetochores were stained overnight with undiluted primary anti-centromere antibody at 37 °C followed by 1:20 diluted FITC-conjugated secondary antibody at 37 °C for 2 h. Nuclei were counterstained with Hoechst 33,258 (5 µg/mL, 3 min). The experiment was performed one time. Per compound, 200 micronuclei were evaluated for kinetochore signal presence (Fig. 3). The evaluation was performed with a Keyence BZ-X800 microscope. The slides used in kinetochore evaluation were also evaluated for the number of induced micronuclei as described in the above paragraph cytokinesis‑block micronucleus assay.

### Tubulin visualization

For tubulin visualization, cells were treated with CBG 30 µM, CBD 15 µM, CBC 15 µM, CBDV 40 µM, and CBN 15 µM. The positive control was vincristine sulphate 0.0121 µM, and the solvent control was DMSO. Cells were treated for 6 h and then harvested. Slides were prepared with cytospin centrifugation. The cells were fixed in ice-cold methanol for 2 h. Following fixation, cells were permeabilized with 0.1% Tween20 in 1 × phosphate-buffered saline (PBS) for 5 min. Next, the anti-α-tubulin antibody was applied in 1:50 dilution. Antibody was diluted with 5% FCS in 1 × PBS. The antibody was incubated in a humid chamber at 4 °C for 24 h. Afterwards, the antibody was removed by washing slides twice with 0.5% Tween20 in 1 × PBS for 5 min. Nuclei were counterstained with Hoechst 33,258 (5 µg/mL, 3 min). The visualization was performed with a Keyence BZ-X800 microscope.

### Cell cycle analysis

The cells were treated with CBG 30 µM, CBD 15 µM, CBC 15 µM, CBDV 40 µM, and CBN 15 µM for 6 or 24 h. The positive control was vincristine sulphate in a concentration of 0.0121 µM for 6 h of exposure and 0.0061 µM for 24 h. The solvent control was DMSO. Furthermore, experiments with the addition of S9 mix were performed with CBDV. In these experiments, cells were treated with CBDV (40 µM) with or without 0.25% S9 mix for 6 h. DMSO with or without the S9 mix was used as control, and the positive control was the same as mentioned above. After the treatment, cells were collected, washed twice with phosphate buffered saline (PBS) and fixed with 70% ethanol for 30 min on ice. Following each described step above, cells were centrifuged at 1000 rpm, 5 min, and 4 °C. Lastly, cells were resuspended in 1% bovine serum albumin (BSA) in PBS solution and stained with DAPI in a final concentration of 1 µg/mL for 30 min on ice. The cell distribution in different cell cycle phases was analyzed with MACSQuant® Analyzer 16 Flow Cytometer using a 450/50 nm filter and MACSQuantify™ Software 2.13 (Miltenyi Biotec, Bergisch Gladbach, Germany). For each sample, 20 000 events were analyzed. Four independent experiments were performed for 6 h of treatment without metabolic activation. Three independent experiments were done for 24 h of treatment without metabolic activation and 6 h of treatment with metabolic activation.

### Statistical analysis

All results are expressed as mean ± standard deviation (SD) from at least three independent experiments except kinetochore labelling results, which are from a one-time experiment. The data were analyzed, and the graphic was drawn with GraphPad Prism version 8 (GraphPad Software, LaJolla, USA). The CBMN and mitotic disturbance assay results were analyzed by one-way ANOVA followed by Dunnett's multiple comparisons tests. The cell cycle analysis results were analyzed by two-way ANOVA followed by Dunnett's multiple comparisons test. The statistical assessment was based on comparing results obtained from cells treated with test compounds and those obtained from cells treated with solvent control. The t-test was used for CBMN experiments with or without the S9 mix to determine the statistical differences between the groups after treating the cells with compounds. The multiple *t* tests were utilized for cell cycle analysis experiments with or without the S9 mix to check the significance between groups after treating the cells with solvent control or compounds. The results were considered significant when the *p* value was < 0.05. The BMD50 estimation was obtained from the micronucleus data using the EFSA web tool for Bayesian BMD analysis, which uses the R-package [BMABMDR] version 0.0.0.9083 for the underlying calculations.

## Results

### Cytokinesis‑block micronucleus (CBMN) assay

**Fig. 4:**
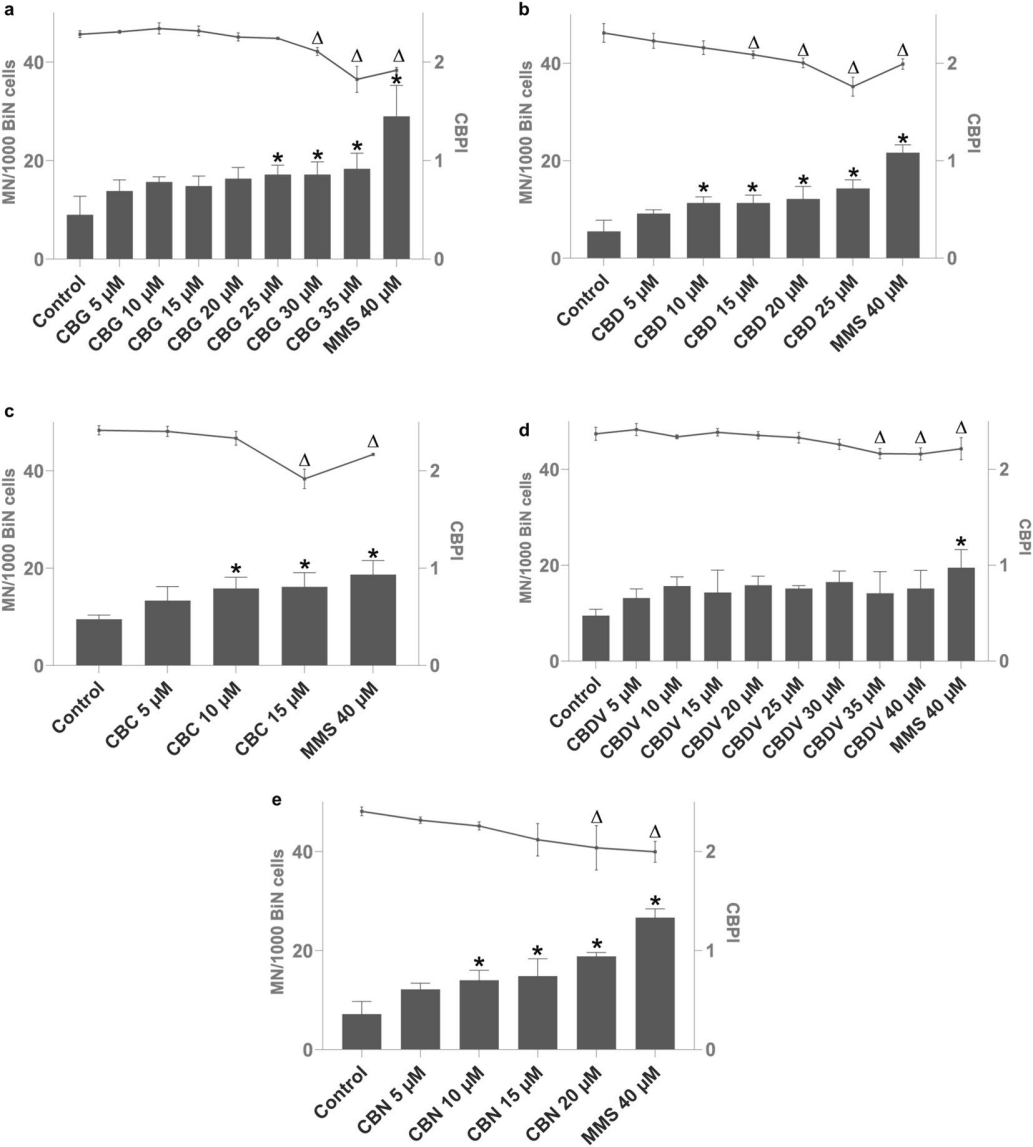
Micronucleus induction (columns) and proliferation index (CBPI; line) in TK6 cells after 4 h of treatment with cannabigerol (CBG) (**a**), cannabidiol (CBD) (**b**), cannabichromene (CBC) (**c**), cannabidivarin (CBDV) (**d**), and cannabinol (CBN) (**e**). The positive control was methyl methane sulfonate (MMS), and the control was solvent, DMSO. *p < 0.05 vs. corresponding control for MN/1000 BiN, Δ*p* < 0.05 vs. corresponding control for CBPI. *MN* micronucleus, *BiN* binucleated cells, *CBPI* cytokinesis-block proliferation index. Results are displayed as mean ± standard deviation (SD) from three independent experiments

**Fig. 5:**
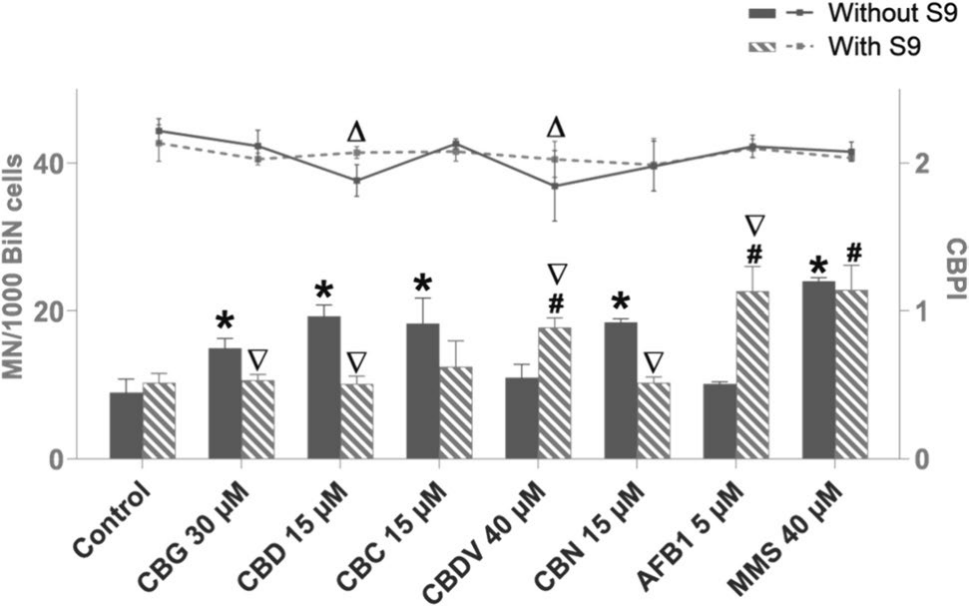
Micronucleus induction (columns) and proliferation index (CBPI; line) in TK6 cells after 4 h of treatment with cannabigerol (CBG), cannabidiol (CBD), cannabichromene (CBC), cannabidivarin (CBDV), and cannabinol (CBN) and presence or absence of metabolic activation. The positive controls were methyl methane sulfonate (MMS) and aflatoxin B1 (AFB1); the control was solvent DMSO. *p < 0.05 vs. corresponding control for MN/1000 BiN without metabolic activation, #*p* < 0.05 vs. corresponding control for MN/1000 BiN with metabolic activation. Δ*p* < 0.05 vs. corresponding control for CBPI without metabolic activation., **∇***p* < 0.05 vs. respective dose without metabolic activation for MN/1000 BiN cells. *MN* micronucleus, *BiN* binucleated cells, *CBPI* cytokinesis-block proliferation index. Results are displayed as mean ± standard deviation (SD) from three independent experiments

The tested cannabinoids CBG, CBD, CBC, and CBN, except CBDV, yielded a significant induction of micronuclei in TK6 cells with at least one concentration after 4 h of treatment (Fig. 4). All tested cannabinoids significantly reduced cell proliferation (indicating cytotoxicity) with at least at one concentration after 4 h of treatment (Fig. 4). Therefore, the chosen dose-ranges covered doses from non-toxic to slightly toxic for all cannabinoids. Determination of BMD showed that BMD50 was similar for CBD, CBC and CBN (8.7; 9.8; 8.4 µM), but about twofold higher for CBG and CBDV (20.5; 15.3 µM) (Table 1). The cannabinoids were also tested with an S9 mix at one concentration that had shown an increase of micronuclei after 4 h without metabolic activation, apart from CBDV, for which the highest tested concentration was chosen. The addition of the metabolic enzymes caused a significant reduction in the number of micronuclei for CBG, CBD, and CBN. For CBC, the number of micronuclei was reduced as well, but not significantly. The combination of CBDV and metabolic enzymes caused a significant rise in micronuclei frequency (Fig. 5).

**Table 1:** BMD50 estimation of cannabigerol (CBG), cannabidiol (CBD), cannabichromene (CBC), cannabidivarin (CBDV), and cannabinol (CBN)

	4 h BMD50 (µM)	BMDL (10th percentile, µM)	BMDU (90th percentile, µM)
CBG	20.5	3.9	64.1
CBD	8.7	2.1	21.1
CBC	9.8	2.5	34.5
CBDV	15.3	1.4	102.9
CBN	8.4	2.7	16.9

The BMD50 estimation, which indicates a 50% increased relative genotoxicity compared to controls, was obtained from the micronucleus data shown in Fig. 4 using Bayesian BMD analysis. The data are presented as the average BMD50 and the 10th and 90th percentiles of the estimation

### Mitotic disturbance assay

**Fig. 6:**
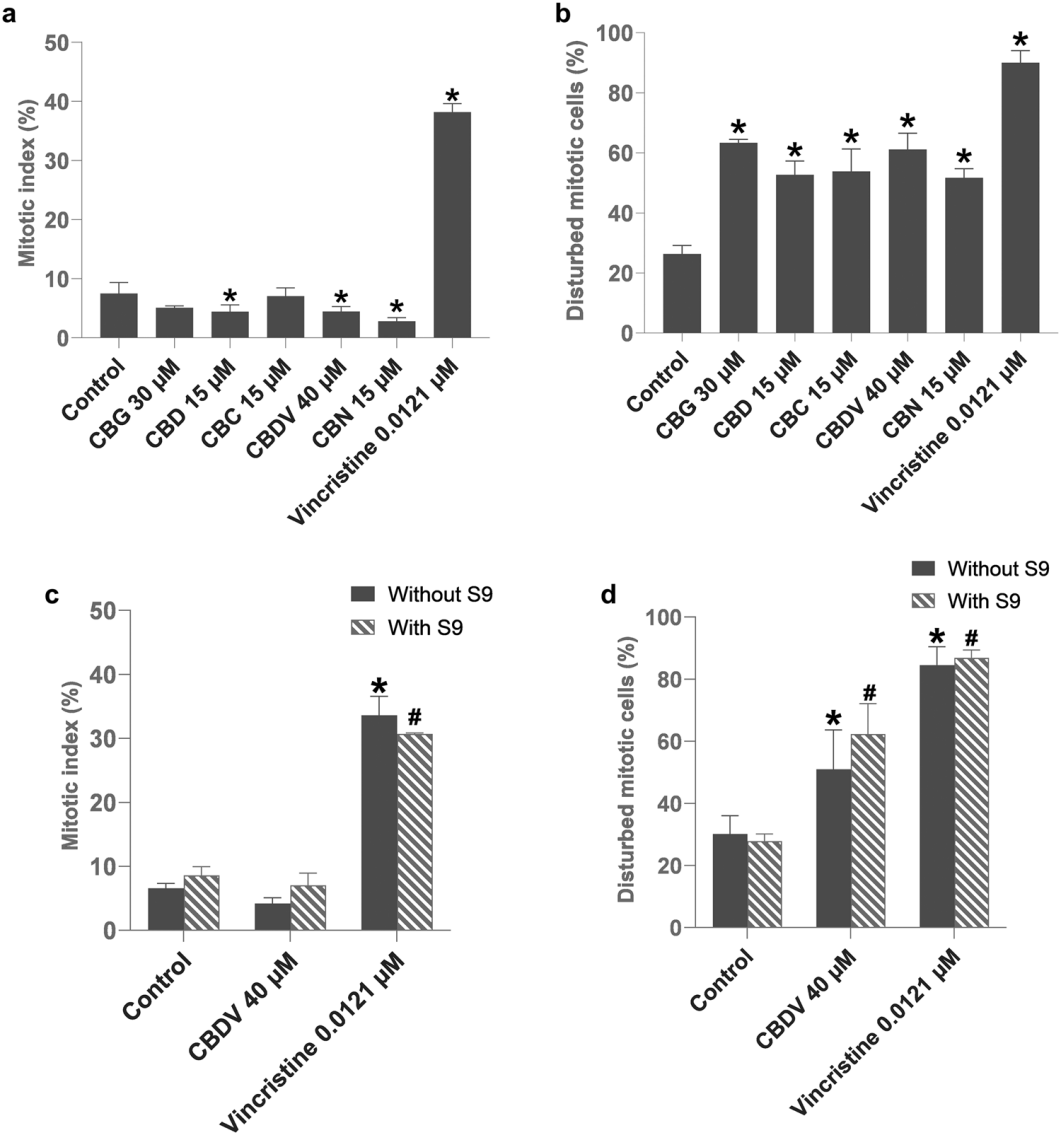
Mitotic index (**a**, **c**) and disturbed mitoses (**b**, **d**) in TK6 cells after 6 h of cannabinoid treatment. Cells were either treated with cannabigerol (CBG), cannabidiol (CBD), cannabichromene (CBC), cannabidivarin (CBDV), and cannabinol (CBN) (**a**, **b**) or treated with only CBDV in the absence or presence of metabolic enzymes (**c**, **d**). The control was solvent DMSO, and the positive control was vincristine. *p < 0.05 vs. corresponding control without metabolic activation, #*p* < 0.05 vs. corresponding control with metabolic activation. The results are represented as mean ± standard deviation (SD) from three independent experiments

The evaluation of mitoses after treatment of TK6 cells with cannabinoids in the absence of metabolic activation is shown as a mitotic index and percentage of disturbed mitoses. Cells were treated with one cannabinoid, CBDV, also in the presence of metabolic activation, as this compound showed an increase of micronuclei in combination with the S9 mix (Fig. 6). CBD, CBDV and CBN significantly decreased the mitotic index compared to solvent control (Fig. 6a). CBDV in combination with S9 mix did not cause a significant alteration of the mitotic index.

In addition, there was no significant difference in mitotic index between CBDV without and with metabolic activation (Fig. 6c). However, all tested cannabinoids caused a significant increase of disturbed mitotic figures compared to the control (Fig. 6b), including CBDV in the absence or presence of metabolic enzymes with the latter being slightly but not significantly higher (Fig. 6d).

### Kinetochore analysis of micronuclei

Using anti-centromere antibody staining, micronuclei were analyzed for the presence of kinetochores (Table 2). While 31% of the control micronuclei exhibited a signal, 41 to 48.5% of the micronuclei in cannabinoid treated and 70% of micronuclei in vincristine treated cells showed a signal. After subtraction of the basal level of micronuclei, the percentage of kinetochore positive induced micronuclei was 61.5% for CBG, 63.7% for CBD, 69.7% for CBC, 69.8% for CBDV, and 63.3% for CBN.

**Table 2:** Kinetochore positive micronuclei (MNi) in human lymphoblastoid TK6 cells treated with cannabigerol (CBG) 30 µM, cannabidiol (CBD) 15 µM, cannabichromene (CBC) 15 µM, cannabidivarin (CBDV) 40 µM, and cannabinol (CBN) 15 µM for 4 h

	MNi	Kinetochore ( +) MNi		Kinetochore ( +) induced MNi^a^
	*N*	*N*	%	%
Control	200	62	31	0
CBG 30 µM	200	87	43.5	61.5
CBD 15 µM	200	94	47	63.7
CBC 15 µM	200	97	48.5	69.7
CBDV 40 µM	200	82	41	69.8
CBN 15 µM	200	92	46	63.3
MMS 40 µM	200	76	38	41.9
Vincristine 0.0121 µM	200	140	70	^b^

The control was solvent DMSO, and the positive controls were methyl methane sulfonate (MMS) and vincristine. The data are presented as number of counted micronuclei, number and percentage of kinetochore positive micronuclei and percentage of kinetochore positive induced micronuclei.

^a^_Kinetochore (+) induced MNi= treatment – control_

^b^_Vincristine could not be evaluated for the percentage of induced micronuclei because the binucleated cells could not be identified clearly._

### Tubulin visualization

**Fig. 7:**
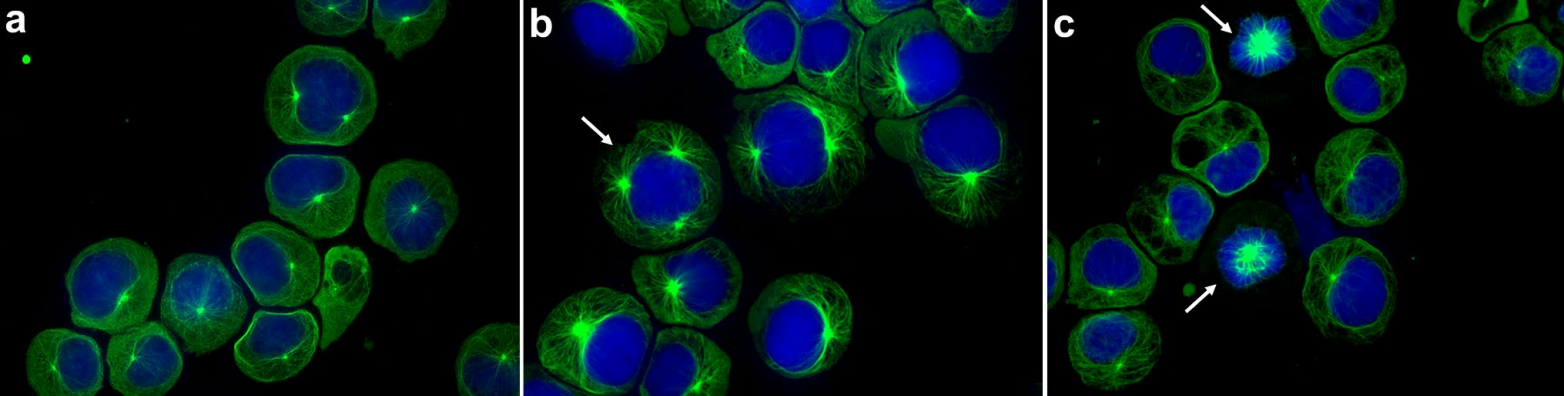
Representative images of tubulin visualization. Nuclei (blue) were stained with Hoechst 33,258, and tubulin (green) with monoclonal anti-α-tubulin antibody. Tubulin distribution in the cells is shown for control (**a**) and cannabinoids (**b**, **c**). Image examples are given for CBG 30 µM (**b**) and CBD 15 µM (**c**). The arrows indicate multipolar tubulin aggregation (b) and monopolar tubulin aggregation (**c**)

Using an anti-α-tubulin antibody, tubulin distribution in the cells was observed. In the cannabinoid-treated cells (CBG 30 µM, CBD 15 µM, CBC 15 µM, CBN 15 µM, Fig. 7), the presence of multipolar tubulin aggregation (Fig. 7b), and monopolar tubulin aggregation (Fig. 7c) was observed more frequently when visually compared to control (not quantified as numbers).

### Cell cycle analysis

**Fig. 8:**
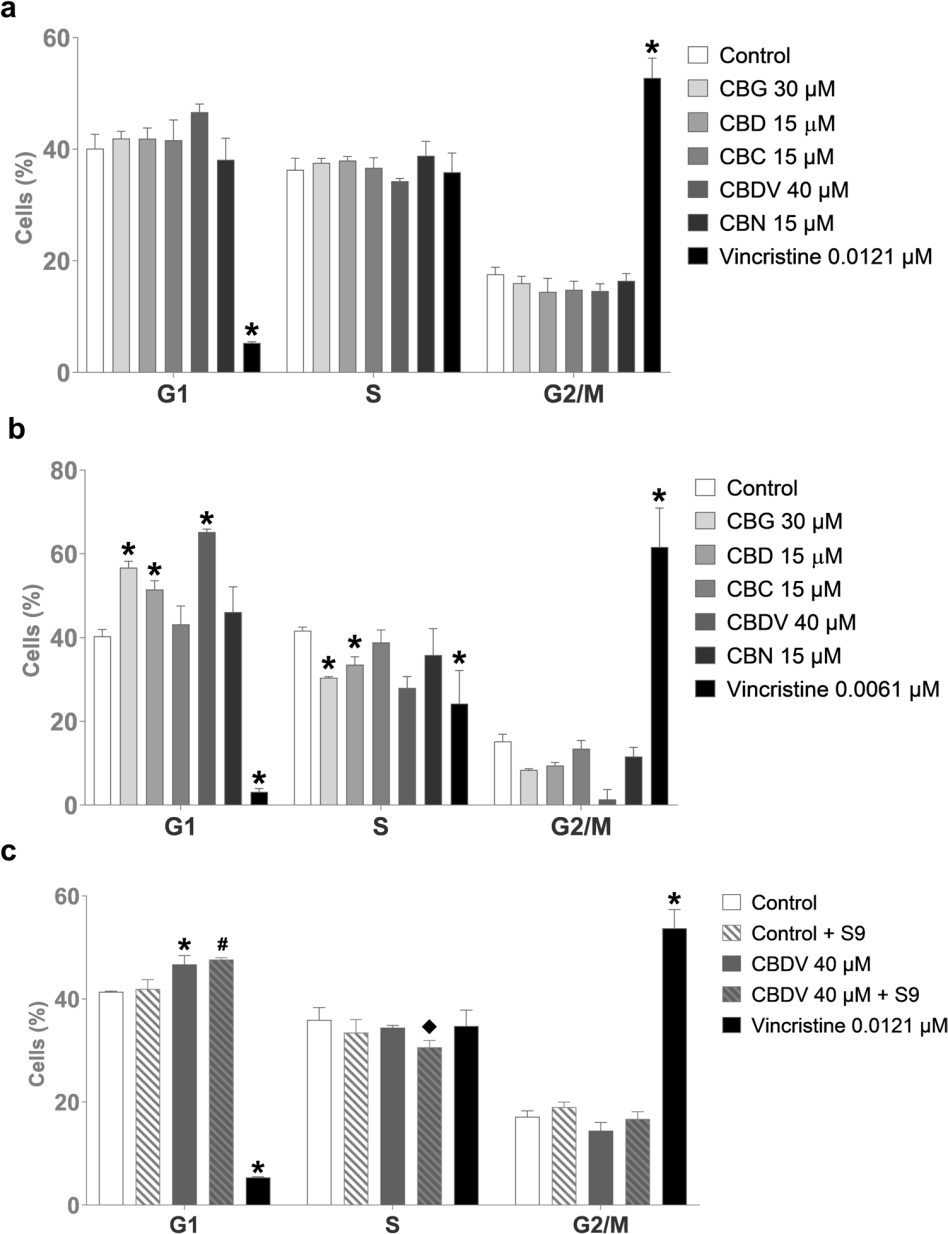
Cell cycle distribution of TK6 cells treated with cannabigerol (CBG), cannabidiol (CBD), cannabichromene (CBC), cannabidivarin (CBDV), and cannabinol (CBN) after 6 h of treatment (**a**), after 24 h of treatment (**b**) and after 6 h of treatment with only CBDV in the absence or presence of metabolic enzymes (**c**). The control was solvent DMSO, and the positive control was vincristine. *p < 0.05 vs. corresponding control without metabolic activation, #*p* < 0.05 vs. corresponding control with metabolic activation, **◆***p* < 0.05 vs. respective dose without metabolic activation. The results are represented as mean ± standard deviation (SD) from four independent experiments for 6 h of treatment and three independent experiments for 24 h of treatment and 6 h of treatment with metabolic activation

Cell cycle analysis of TK6 cells treated with either CBG, CBD, CBC, CBDV, or CBN for 6 h showed no significant changes in the normal cell cycle distribution. However, there was a slight non-significant rise in cell percentage in the G1 phase for CBDV treatment compared to the control (DMSO) (Fig. 8a). Increasing the treatment to 24 h resulted in the accumulation of cells in the G1 phase when treated with CBG, CBD, and CBDV (Fig. 8b). In the case of CBDV, the cell cycle distribution of TK6 cells was also analyzed after addition of metabolic enzymes (S9). TK6 cells treated with CBDV in the absence or presence of metabolic enzymes showed cell accumulation in the G1 phase after 6 h compared to the corresponding control. Since the S9 mix becomes toxic during extended treatment durations, CBDV with S9 could not be tested after 24 h. Furthermore, CBDV incubated with liver enzymes caused a minor reduction in cell percentage in the S phase compared to CBDV without liver enzymes (Fig. 8c).

## Discussion

As the worldwide consumption of *Cannabis* dietary products increases constantly, it is critical to determine whether the compounds present in *Cannabis* have beneficial or adverse potential. In this study, we have tested the *Cannabis*-derived cannabinoids: cannabigerol (CBG), cannabidiol (CBD), cannabichromene (CBC), cannabidivarin (CBDV), and cannabinol (CBN) to see their effects on cells regarding genotoxicity, mitosis, and cell cycle.

Micronuclei are long-used biomarkers for genotoxicity (Kwon et al. 2020), as their presence indicates defects in the cell repair machinery or accumulation of DNA damages, chromosomal aberrations (Luzhna et al. 2013) or chromosome loss. The micronucleus assay results in the present study show that all tested cannabinoids, except CBDV, caused a mild but significant rise of micronuclei in cells after 4 h of treatment without metabolic activation. The addition of metabolic enzymes to the human lymphoblastoid TK6 cells with cannabinoid treatment led to a detoxification of the cannabinoids, except for CBDV, which showed increased micronuclei formation, i.e. metabolic activation. Publications containing experimental data regarding in vitro genotoxicity of CBG, CBC, and CBN isolates are not currently available, according to our knowledge. However, experimental data for CBD and CBDV are accessible. The publications that have used CBD in in vitro settings showed contrasting results, possibly due to varying concentrations of CBD, different cell materials, experimental setups, and duration of treatment. Russo et al. (2019) found that CBD damaged DNA in the human buccal cell line TR146 after 3 and 24 h, and the addition of liver enzymes increased the genotoxic properties detected in single cell gel electrophoresis (comet assay). We cannot explain the difference regarding metabolic activation of CBD to our results with certainty, but a higher capacity of TR149 for transmembrane transport compared to TK6 cells used here and the different protocol between comet assay and micronucleus assay (with a substance-free expression time after treatment in the latter) may be discussed. Russo et al. (2019) also found a significant induction of micronuclei with CBD in human hepatoma cells after 3 h of treatment. On the contrary, Aviello et al. (2012) published that CBD did not induce DNA damage in the comet assay in colon-derived cells after 24 h of treatment. Furthermore, Henderson et al. (2023) reported negative micronucleus results in human lymphoblastoid TK6 cells with and without metabolic activation after 4 h of treatment and 27 h of treatment without metabolic activation. For the genotoxicity of CBDV, only the study by Russo et al. (2019) was found, which reported that CBDV had the same effect on the cells as CBD. Studies investigating in vitro genotoxicity of either hemp extracts or CBD-rich oils reported negative results in bacterial reverse mutation assay, chromosomal aberration test and micronucleus assay (Marx et al. 2018; Dziwenka et al. 2020, 2023; Clewell et al. 2023). Also, these studies did not see a genotoxic potential of hemp extracts or CBD-rich oils in vivo. Evidence about in vivo genotoxicity of cannabinoid constituents exists only for CBD. European Medicine Agency (EMA) (2019) published negative results in micronucleus and comet assay of bone marrow or liver of rats after CBD treatment, while Zimmerman and Raj (1980) reported induction of micronuclei in the bone marrow of mice.

The presence of micronuclei in cannabinoid-treated cells raised the question of whether disturbance of mitosis occurs in those cells, as it is known that errors during cell division can lead to the formation of micronuclei in cells (Beedanagari 2017). Our results showed that cannabinoids interfere with mitosis, seen as an increased percentage of disturbed mitotic figures. Older studies have shown that olivetol, a typical ring structure of cannabinoids, can induce segregation errors of chromosomes in human peripheral lymphocytes (Morishima et al. 1976), as well as tetrahydrocannabinol (THC) (Henrich et al. 1980). Shinohara et al. (1983) saw that THC intervenes in the process of meiosis by preventing ova cells from finishing the first cleavage division.

Likewise, Zimmerman et al. (1978) reported that THC impairs spermatogenesis by inducing cytogenetic abnormalities, which was also stated for CBD and CBN. A possible mechanism of mitotic disturbance is that cannabinoids disrupt microtubular formation or structure, as it was previously indicated that THC, CBD, and CBN had obstructed tubulin synthesis in Chinese hamster ovary and rat adrenal phaeochromocytoma cells (Tahir et al. 1991, 1992). Furthermore, THC was shown to affect microtubule polymerization and stability in the brains of mice and rats (Tortoriello et al. 2014; Gholami et al. 2020). In fact, we also observed an increased frequency of multipolar or monopolar mitotic spindle formation, supporting the idea of disturbed tubulin dynamics. Opposite to that, actin filament distribution did not change and also, chromosome condensation was not altered visibly after cannabinoid treatment (not shown).

The micronucleus content can be differentiated by kinetochore staining. Kinetochore-positive micronuclei consist mainly of whole chromosomes, whereas the induction of kinetochore-negative micronuclei indicates a substance's potential chromosome-breaking ability (Manshian et al. 2013). Kinetochore staining revealed that the vast majority of the induced micronuclei were positive, meaning cannabinoids predominately are likely to induce the segregation of whole chromosomes into micronuclei, further supporting mitotic disturbance as a major driver in cannabinoid-induced micronucleus formation.

Considering that errors in the mitotic process can trigger either cell cycle arrest, senescence, or apoptosis (Levine and Holland 2018), we conducted a cell cycle analysis of cannabinoid-treated human lymphoblastoid TK6 cells. Cannabinoid treatment for 6 h did not induce a change in cell cycle distribution. This is in agreement with the fact that we did not observe the increased overall number of mitoses despite an increase of disturbed mitotic figures. However, 24 h of treatment led to a slight, but significant accumulation of cells in the G1 phase when incubated with CBG, CBD, or CBDV. Previous reports indicate that CBG changes the cell cycle by arresting cells in the G0/G1 phase, which correlates with a decrease of cell percentage in the S phase in human mesothelioma, cholangiocellular carcinoma and ovarian carcinoma cell lines after 24 h of treatment (Viereckl et al. 2022; Colvin et al. 2022; Sooda et al. 2023). Likewise, after 24 h of treatment, CBD caused G1 phase cell cycle arrest by upregulating expression of p53 and reducing levels of CDK2 and cyclin E in human gastric cancer, Sertoli and Leydig cells (Zhang et al. 2019; Li et al. 2022, 2023). We have not found any reported results of CBDV's impact on the cell cycle, except one publication by Lourenço et al. (2023), which reported that CBDV induced cell cycle arrest of adult neural stem/progenitor cells and stimulated their differentiation into neurons after 7 days of exposure. However, they did not indicate in which phase of cell cycle progression was disturbed. CBN was also reported to trigger cell cycle arrest in either the G0/G1 phase or S phase, depending on the cell line, by downregulating CDK1 and CDK2 and cyclin E (Zhong et al. 2023). This effect was not observed in our study. In addition, we did not observe a change in cell cycle distribution after treatment with CBC, which increased the number of cells in the S phase when combined with THC (Anis et al. 2021).

Some aspects of the induction of micronuclei by cannabinoids remain to be determined. For example, the dose–response curve seems to show a saturation type curve, and it is an open question whether mixtures of cannabinoids, as they typically occur in plant extracts and plant-derived products, might exert additive effects and, therefore, increase the maximally possible micronucleus induction. Another open question is why CBG, CBD, CBC, and CBN induced micronuclei formation without metabolic activation, but CBDV induced micronuclei formation after metabolic activation. One of the possible explanations could be the differences in their chemical structures. The cannabinoids are meroterpenoids with a resorcinyl core and a para-positioned isoprenyl, alkyl, or aralkyl side chain (Hanuš et al. 2016).

All cannabinoids from *Cannabis sativa* have an alkyl side chain (Blatt-Janmaat and Yang 2021), and depending on alkyl side chain length, they can be divided into several classes: orcinoids, which contain one carbon; varinoids, which contain three carbons; and olivetoids, which contain five carbons. CBG, CBD, CBC, and CBN belong to the olivetoid class, while CBDV belongs to the varinoid class (Gülck and Møller 2020). Previous structure–activity relationship studies on cannabinoids have identified the lipophilic alkyl side chain as the most critical group for the biological potency of cannabinoids (Thakur et al. 2005). Olivetoids (CBG, CBD, CBC, and CBN) and varinoids (CBDV) also have different susceptibilities for biotransformation (McGilveray 2005; Havlasek et al. 2023). However, the genotoxic metabolite of CBDV is not known at this point. The genotoxicity mechanism of cannabinoids could not be elucidated entirely here. We clearly observed a higher prevalence of abnormal mitosis as well as different tubulin patterns and induction of kinetochore-containing micronuclei. Therefore, it could be hypothesized that cannabinoids are genotoxic via disturbance of mitosis by negatively affecting tubulin, which predominantly results in the segregation of whole chromosomes into micronuclei observed with kinetochore staining. However, interference with tubulin aggregation or spindle disassembly commonly causes elevated mitotic indices, which was not observed here, meaning that the cells were either not arrested in mitosis or that many did not reach mitosis. This is supported by the cell cycle analysis to some extent, where an exposure duration as that used in the micronucleus assay did not cause any alterations, and more prolonged exposure led to an increase of G1 phase cells. The type of disturbance seemed similar for all tested cannabinoids, but the effective concentrations varied.

The effects were seen at micromolar concentrations of cannabinoids, which are higher than concentrations found in consumers' blood. We have not found any reported results of blood levels of CBG and CBN after oral dosing. However, CBG and CBN are often found in consumer's blood after inhalation. The mean maximum concentration (C_max_) of CBG and CBN in the blood of frequent *Cannabis* smokers were 6.9 µg/L (21.8 nM) and 11.6 µg/L (37.4 nM), respectively (Newmeyer et al. 2016). For CBD, the mean plasma C_max_ after administration of an oral solution of CBD in healthy adults was 335.4 µg/L (1.1 µM), with an increase to 1628 µg/L (5.2 µM) following a high-fat meal (Taylor et al. 2018). Hurley et al. (2022) reported that the mean plasma C_max_ for CBDV as epilepsy treatment in children was 7.4 µg/L (25.8 nM), and for CBC, it was reported that a mean C_max_ concentration was 6.6 µg/L (21.0 nM) in participants' plasma after consuming medical cannabis oil (Peters et al. 2022). Thus, the positive responses in our in vitro study were achieved with about 1000-fold higher concentrations than the observed blood levels.

Altogether, the selected cannabinoids (except CBDV) induced micronuclei at concentrations several orders of magnitude higher than those found in human plasma, and the activity was reduced after the application of a metabolic activation system (S9). It may be concluded that, therefore, oral ingestion poses no genotoxic risk for human consumption. However, in the context of preparations in oil, which are supposed to be kept in the mouth for some time, absorbance through the buccal mucosa is possible and systemic distribution occurs without a liver passage and liver enzyme inactivation. Furthermore, the situation is different for CBDV because this cannabinoid became moderately genotoxic in the presence of metabolic enzymes, although at micromolar concentrations. However, this does show that unknown cannabinoids may be metabolically activated, indicating a need for further analytical identification and investigation of natural cannabinoids.

Since the effects may be mediated through disturbance of the mitotic machinery rather than direct DNA damage, there may be a dose range below which there is no adverse effect, which would also indicate possible safe ingestion of low amounts of cannabinoids.

In conclusion, this study provides insights into the genotoxicity of some selected cannabinoids. While metabolic inactivation occurred for most of them, metabolic activation was detected for CBDV. The investigated cannabinoids were chosen according to the abundance of cannabinoids in *Cannabis sativa*. There are many more cannabinoids present at lower concentrations in *Cannabis sativa* and in *Cannabis*-derived extracts, preparations, or dietary products, which—in light of our findings—need to be investigated regarding their genotoxic potential. Cannabinoid concentrations used in this study are probably not achievable for a consumer. Nevertheless, data gaps need to be filled for proper risk assessment for consumer safety.

## Data Availability

Available upon request.
